# Beyond auxin responses: a miR160-ARF4 module regulates the biogenesis of medicinal saponins

**DOI:** 10.1093/plphys/kiag731

**Published:** 2026-10-01

**Authors:** Avilash Singh Yadav

**Affiliations:** Assistant Features Editor, Plant Physiology, American Society of Plant Biology; Department of Biology, College of Natural Sciences, University of Massachusetts Amherst, Amherst, MA 01003, United States

Among multiple strategies that plants must adopt to ensure survival fitness, one strategy involves the production of specialized compounds called secondary metabolites. These compounds may not directly regulate growth; however, they play a major role in defense responses and stress adaptation (Jeyasri et al. 2023). Plants produce more than 200,000 secondary metabolites, many of which are used to produce medicines to treat malaria, heart diseases, and cancer (Yonekura-Sakakibara and Saito 2009; Khanam et al. 2025). Yet in many medicinal plants, biosynthesis of these therapeutic compounds can vary based on the developmental stage and environmental conditions (Li et al. 2020). This makes it very difficult to predict and standardize the yield and composition of secondary metabolites for manufacturing pharmaceuticals. Thus, for reliably producing such highly valuable medicinal metabolites, understanding the underlying molecular mechanisms is essential.

*Panax notoginseng*, a perennial herb belonging to the Araliaceae family, is one such medicinal plant that has traditionally been used in Chinese medicine (Xu et al. 2019). The medicinal value of this plant primarily comes from *Panax notoginseng* saponins (PNS), which are dammarane-type tetracyclic triterpenoid glycosides that are highly valued for their use in improving cardiovascular health (Zhang et al. 2008; Wei et al. 2020). Although the PNS biosynthesis pathway is well characterized, and multiple transcription factors (TFs) that regulate this pathway have also been identified, the upstream components that may potentially regulate these TFs remain elusive.

Several TFs are regulated by microRNAs (miRNAs), which are small (20 to 24 nt) regulatory RNAs that bind to complementary sequences in the target transcripts and repress their expression (Borges and Martienssen 2015). Among these miRNA-TF modules, miR160 regulates a conserved subset of AUXIN RESPONSIVE FACTOR (ARF) TFs, which are best known for regulating the expression of auxin responsive genes and modulating plant growth and development (Hao et al. 2022). Interestingly, miR160-ARF modules have also been shown to regulate secondary metabolite biosynthesis in medicinal plants (Guo et al. 2023). This context raises the question as to whether the miR160-ARF module also regulates PNS biosynthesis in *Panax notoginseng*.

In this issue of *Plant Physiology*, Li et al. (Li et al. 2026) characterized the role of the Pn-miR160a-PnARF4 module in regulating the biosynthesis of pharmacologically active PNS. Initially, the authors found that a broad set of PNS biosynthesis genes was induced when *Panax notoginseng* cells were treated with methyl jasmonate (MeJA), a hormone commonly used to stimulate secondary metabolite production. Based on small RNA sequencing analysis, the authors identified several miRNAs representing 30 miRNA families across control and MeJA-treated samples. The abundance of 8 distinct miRNAs was significantly altered after MeJA treatment, coinciding with the induction of saponin biosynthesis genes. Specifically, miR12150, miR172b-5p_7, miR6300, miR394a, and miR403a were downregulated, while miR397b_3, miR399a-3, and miR160a were upregulated in MeJA-treated samples. These findings were validated through RT-qPCR as well, indicating that these miRNAs could potentially regulate saponin biosynthesis in *Panax notoginseng*.

Out of these 8 candidate miRNAs, miR160a and miR399-3p seemed particularly interesting, since they belong to highly conserved miRNA families (Zhao et al. 2025). Prediction analysis using psRNATarget (Dai et al. 2018) suggested that Pn-miR160a targets ARF transcription factors. Since the role of the miR160a-ARF module in plant development, stress responses, and secondary metabolism is relatively well established (Hao et al. 2022; Guo et al. 2023), the authors prioritized the investigation of the role of Pn-miR160a in PNS production.

If Pn-miR160a does play a role, would altering Pn-miR160a abundance affect PNS biosynthesis? To test this, the authors generated *Panax notoginseng* cell lines that either overexpressed Pn-miR160a (Pn-miR160a-OE) or expressed a short tandem target mimic (STTM) that reduces Pn-miR160a abundance (Pn-miR160a-STTM). Briefly, STTMs are artificial constructs that bind to target miRNAs with high specificity, thus compromising the ability of the miRNAs to regulate endogenous transcripts (Yan et al. 2012). Among genes encoding key enzymes in the saponin biosynthesis pathway, the authors identified that the expression of 3 genes, namely *PnSE*, *PnCYP716A47*, and *PnCYP716A53v2*, was strongly upregulated in Pn-miR160a-OE lines. Conversely, these genes were also downregulated in Pn-miR160a-STTM lines, suggesting that Pn-miR160a selectively regulates these specific nodes of the saponin biosynthesis pathway (Fig. 1). Consistent with the observed changes in gene expression, total saponin content in the Pn-miR160a-OE lines increased by approximately 1.78-fold relative to the wild type, while Pn-miR160a-STTM lines could accumulate only 57.14% of the saponin levels in the wild type. The authors also observed a similar trend across 5 major saponins: notoginsenoside R1 and ginsenoside Rg1, Re, Rb1, and Rd. In summary, these findings clearly demonstrate that Pn-miR160a positively regulates PNS biosynthesis.

**Figure 1:**
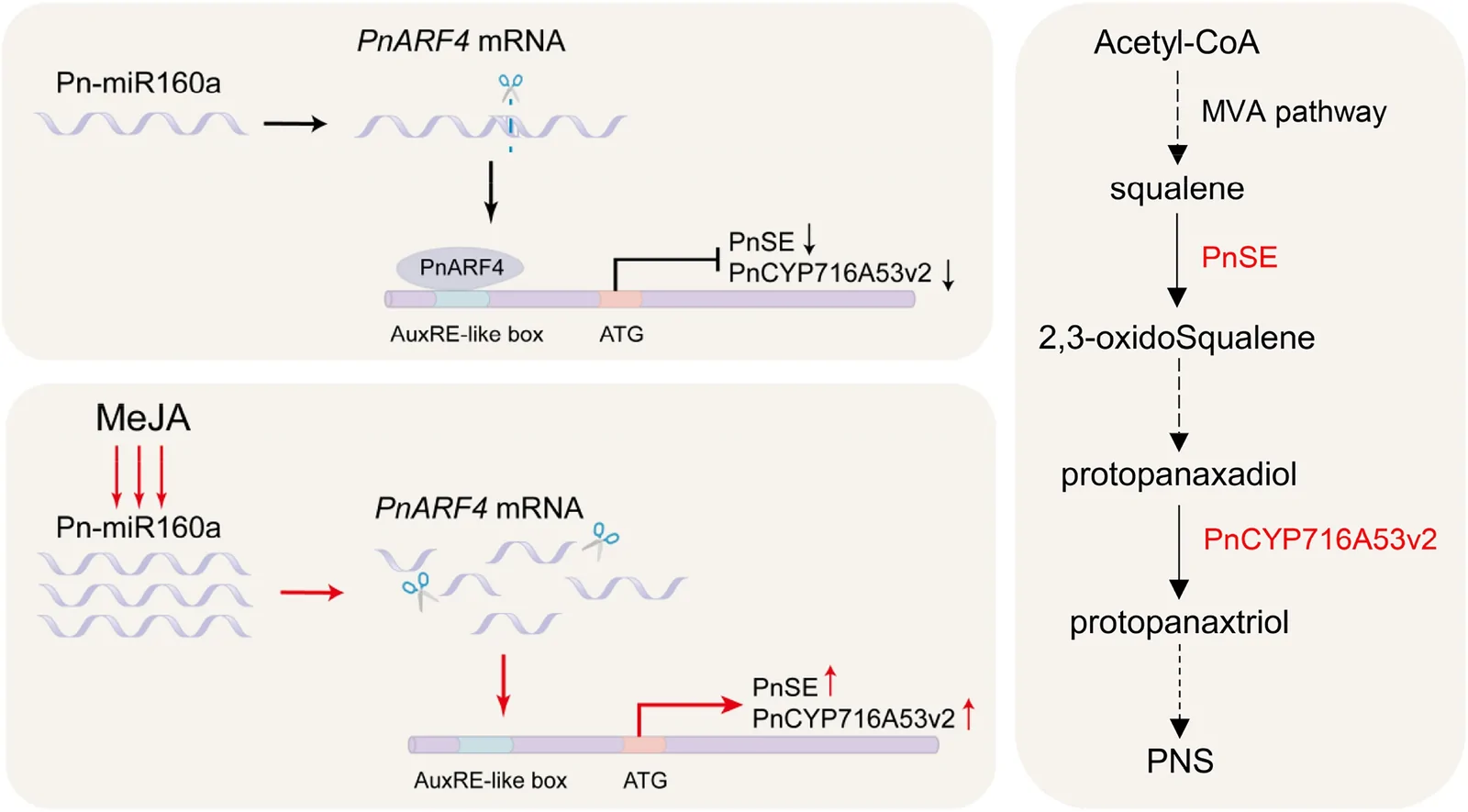
Schematic showing how the Pn-miR160a-PnARF4 module regulates saponin biosynthesis *Panax notoginseng*. (Top left panel) Under normal conditions, low Pn-miR160a abundance allows for PnARF4 accumulation. Subsequently, PnARF4 directly binds to the AuxRE-like motifs within the promoters of saponin biosynthesis genes *PnSE* and *PnCYP716A53v2* to repress their expression. (Bottom left panel) Exogenous MeJA treatment induces *Pn-miR160a* expression, following which Pn-miR160a binds to and degrades *PnARF4* transcripts. As a result, reduced PnARF4 abundance relieves the transcriptional repression of *PnSE* and *PnCYP716A53v2*, thus promoting saponin biosynthesis. (Right panel) Important steps of the PNS biosynthesis pathway are shown. PnSE catalyzes the conversion of squalene to 2,3-Oxidosqualene, while PnCYP716A53v2 catalyzes the conversion of protopanaxadiol to protopanaxtriol. Reproduced from (Li et al. 2026).

The authors then asked whether Pn-miR160a regulates PNS biosynthesis through their target ARFs, and if so, precisely which ARF. Among the 30 *PnARF* genes identified in the *Panax notoginseng* genome, a total of 4 genes were found to harbor putative Pn-miR160a binding sites. Among them, only *PnARF4* showed the expected inverse expression pattern, where *PnARF4* was downregulated in Pn-miR160a-OE lines and upregulated in Pn-miR160a-STTM lines. Using luciferase and GUS assays, the authors also confirmed that Pn-miR160a directly targets *PnARF4* transcripts to repress *PnARF4* expression (Fig. 1). In line with the hypothesis that *PnARF4* negatively regulates PNS biosynthesis, the expression levels of *PnSE*, *PnCYP716A47*, and *PnCYP716A53v2*, which were upregulated in Pn-miR160a-OE lines, were significantly downregulated in lines overexpressing *pnARF4* (*PnARF4*-OE) (Fig. 1). These transcriptional changes were accompanied by an average 42.85% reduction in the total PNS content in *PnARF4*-OE lines relative to the wild type. Overall, these observations are consistent with the idea that Pn-miR160a promotes saponin biosynthesis by directly repressing *PnARF4*.

To further investigate the underlying molecular mechanism, the authors tested whether PnARF4 directly represses PNS biosynthesis genes. ARFs preferentially bind to AuxRE-like promoter elements (O’Malley et al. 2016). While putative AuxRE-like motifs were identified in the promoters of all 3 major saponin biosynthesis genes, yeast 1-hybrid assays revealed that PnARF4 binds specifically to the promoters of *PnSE* and *PnCYP716A53v2*, and not *PnCYP716A47*. Using dual luciferase assays, the authors experimentally confirmed that PnARF4 directly represses the transcription of PNS biosynthesis genes *PnSE* and *PnCYP716A53v2* (Fig. 1).

Taken together, Li and colleagues (Li et al. 2026) demonstrated that Pn-miR160a promotes saponin biosynthesis in *Panax notoginseng* by repressing the TF ARF4, showing how an auxin-mediated growth regulatory module is recruited to regulate secondary metabolism as well. Since miR160 has been linked to specialized metabolite production across multiple medicinal plants, this study provides additional evidence to show that miR160-ARF modules may have a more broadly conserved role in regulating metabolism (Guo et al. 2023; Li et al. 2026; Xing et al. 2026). It would be interesting to investigate whether the miR160-ARF module also regulates triterpenoid saponin biosynthesis in other *Panax* or Araliaceae species. If so, this opens an exciting avenue to explore how this framework could be leveraged to improve medicinal metabolite production in general.

## Data Availability

There is no new data associated with this News & Views article.

## References

[kiag731-B1] Borges F, Martienssen RA. 2015. The expanding world of small RNAs in plants. Nat Rev Mol Cell Biol. 16:727–741. 10.1038/nrm4085. PMC4948178 26530390

[kiag731-B2] Dai X, Zhuang Z, Zhao PX. 2018. psRNATarget: a plant small RNA target analysis server (2017 release). Nucleic Acids Res. 46:W49–W54. 10.1093/nar/gky316. PMC6030838 29718424

[kiag731-B3] Guo Z et al 2023. Profiling of phytohormone-specific microRNAs and characterization of the miR160-ARF1 module involved in glandular trichome development and artemisinin biosynthesis in Artemisia annua. Plant Biotechnol J. 21:591–605. 10.1111/pbi.13974. PMC9946145 36478140

[kiag731-B4] Hao K et al 2022. Mir160: an indispensable regulator in plant. Front Plant Sci. 13:833322. 10.3389/fpls.2022.833322. PMC8981303 35392506

[kiag731-B5] Jeyasri R et al 2023. Methyl jasmonate and salicylic acid as powerful elicitors for enhancing the production of secondary metabolites in medicinal plants: an updated review. Plant Cell Tissue Organ Cult. 153:447–458. 10.1007/s11240-023-02485-8. PMC10026785 37197003

[kiag731-B6] Khanam S et al 2025. Plant-based secondary metabolites as natural remedies: a comprehensive review on terpenes and their therapeutic applications. Front Pharmacol. 16:1587215. 10.3389/fphar.2025.1587215. PMC12127327 40458805

[kiag731-B7] Li W, Liu C, Qu Y, Liu D, Ge F. 2026. miR160a-ARF4 module regulates the biosynthesis of Panax notoginseng saponins. Plant Physiol. 202:kiag656. 10.1093/plphys/kiag656. 42687763

[kiag731-B8] Li Y, Kong D, Fu Y, Sussman MR, Wu H. 2020. The effect of developmental and environmental factors on secondary metabolites in medicinal plants. Plant Physiol Biochem. 148:80–89. 10.1016/j.plaphy.2020.01.006. 31951944

[kiag731-B9] O’Malley Ronan C et al 2016. Cistrome and epicistrome features shape the regulatory DNA landscape. Cell. 165:1280–1292. 10.1016/j.cell.2016.04.038. PMC4907330 27203113

[kiag731-B10] Wei G et al 2020. Metabolomes and transcriptomes revealed the saponin distribution in root tissues of Panax quinquefolius and Panax notoginseng. J Ginseng Res. 44:757–769. 10.1016/j.jgr.2019.05.009. PMC7655499 33192118

[kiag731-B11] Xing B et al 2026. Csa-miR160 targets transcriptional repressor CsARF18 and modulates auxin-mediated crocin biosynthesis in Crocus sativus L. Plant Physiol. 201:kiag142. 10.1093/plphys/kiag142. 41838799

[kiag731-B12] Xu C et al 2019. Analytical methods and biological activities of Panax notoginseng saponins: recent trends. J Ethnopharmacol. 236:443–465. 10.1016/j.jep.2019.02.035. 30802611

[kiag731-B13] Yan J et al 2012. Effective small RNA destruction by the expression of a short tandem target mimic in Arabidopsis. Plant Cell. 24:415–427. 10.1105/tpc.111.094144. PMC3315224 22345490

[kiag731-B14] Yonekura-Sakakibara K, Saito K. 2009. Functional genomics for plant natural product biosynthesis. Nat Prod Rep. 26:1466–1487. 10.1039/b817077k. 19844641

[kiag731-B15] Zhang YG et al 2008. Panax notoginseng saponins attenuate atherosclerosis in rats by regulating the blood lipid profile and an anti-inflammatory action. Clin Exp Pharmacol Physiol. 35:1238–1244. 10.1111/j.1440-1681.2008.04997.x. 18637019

[kiag731-B16] Zhao X et al 2025. MicroRNAs in plants development and stress resistance. Plant Cell Environ. 48:5909–5929. 10.1111/pce.15546. 40255181

